# Tree responses and temperature requirements in two central Italy phenological gardens

**DOI:** 10.1007/s00484-023-02522-3

**Published:** 2023-08-01

**Authors:** Marco Fornaciari, Silvia Marrapodi, Luigia Ruga, Chiara Proietti, Fabio Orlandi

**Affiliations:** Department of Civil and Environmental Engineering, University of Perugia, Borgo XX Giugno 74, 06121 Perugia, Italy

**Keywords:** Plant phenology, Heat accumulation, GDD, Climate change, Bioindicators

## Abstract

Plants have always been able to adapt to climate change by reacting through various responses, mainly at the phenological level. The aim of this work is to investigate the behavior of specific tree species located in two phenological gardens in central Italy in relation to the temperature increases recorded in recent years. Specifically, four main phenological phases, BBCH_11, BBCH_19, BBCH_91, and BBCH_65, were monitored during a 14-year time period. The data of the weeks corresponding to the first appearance of each phenological phase and the respective heat accumulations for each species were cross-referenced with the meteorological data recorded by the stations in the two considered areas. Based on average temperature, calculated over reference periods, the species were divided by creating “warm” year groups and “cold” year groups so as to better highlight any differences in the behavior of the same species. In addition, a strong correlation was shown between the maximum temperatures in February and the advances of phenological phases BBCH_11 and BBCH_65. Most of the tree species have shown strong adaptation to climate warming, changing the period of occurrence of the phases themselves.

## Introduction

Phenology is defined as “the study of the timing of recurring biological events, the causes of their timing in relation to biotic and abiotic forces, and the correlation between phases of the same or different species” (Lieth 1974). It is a science focused on the observation of the developmental phases of plants in response to seasonal variations and on recording the dates when the phases occur in different environments. The rhythmic responses of vegetation are determined by the genetic characteristics of each plant and by numerous other environmental characteristics (soil, climate, cultural practices, soil and air pollution, phytopathogens, etc.). All the information gathered from phyto-phenological observations can be used in many areas, such as agriculture, forestry, allergology, or tourism. Phenological Gardens are plots of land where appropriately chosen plants are planted and periodically observed to study the effects of climate and seasonal weather on their development. The gardens are useful for obtaining interpretive answers about “peculiar” climatic trends, which in recent years have resulted in considerable variations for both wild and widely cultivated plant species. Specifically, standardized observations and measurements are made within Phenological Gardens on particular plant essences referred to as “Guide Species” or “Indicator Species,” which are sensors of climatic variations and environmental quality, especially air quality, with respect to pollutants. The selected species are widely distributed throughout the country as well as internationally, have different production cycles and staggered flowering from winter to late spring. Phenological Gardens present throughout the Italian territory follow a common international protocol regarding planting methods, list of plants to be considered, observation methodology, and archiving and processing of phenological data (Renner and Chmielewski 2022).

Through phenology, it has been shown that the timing of the developmental stages of plant species has undergone significant changes as a result of the relatively modest levels of climatic warming observed to date. Phenology studies have been very important to show, through concrete examples, that climate change has already happened and is real. All plant species can be considered as potential bioindicators when properly placed in their ecosystem; phenology shows alterations caused by various environmental factors, applied to biological modeling and measurement of climate change (Gordo and Sanz 2010; Parmesan 2007; Zhao and Schwartz 2003). Climate change can affect the vegetative-reproductive cycle of plant species to a greater or lesser extent. Plants shift their phenology in the direction predicted on the basis of current climate changes (Parmesan and Yohe 2003). Global warming levels have been associated with the risk of increased water scarcity in drylands, vegetation loss, and decreased crop yields with consequent risks for food supply (IPCC 2019).

To enable the development and survival of tree species in different environments, two processes are crucial, genetic diversity and phenotypic plasticity (Pigliucci et al. 2006; Vitasse et al. 2010). The first improves adaptive capacities in a new environment, the second is one of the most significant ways in which plants respond and cope with climate change by managing to persist in their environment (Sultan 2004; Pigliucci et al. 2006; Ghalambor et al. 2007; Valladares et al. 2006; Vitasse et al. 2010). Species distribution is influenced by phenotypic plasticity, which is determined by temperature (T) and can be assessed by considering tree-specific traits such as phenology, growth, and frost resistance (Chuine and Beaubien 2001; Vitasse et al. 2010). In a study by Vitasse et al (2010), the leaf phenology of two dominant tree species in European areas, such as *Quercus petraea* and *Fagus sylvatica*, was evaluated to understand the behavior of leaves in relation to temperature. The results showed that for each degree increase, the date of leaf unfolding was advanced in both species with an average shift of 5 and 7 days, respectively. From this, it appears that both tree species possess high phenological plasticity given the ability to respond immediately to variations of temperature. In addition, the results of their work suggest that climate warming could lengthen the vegetative period of all oak populations located along a greater altitudinal gradient, compared to low-elevation populations such as beeches, which may instead experience earlier senescence and a shorter vegetative period due to other warming-related changes. Several studies have revealed phenological trends in the Mediterranean area that agree with recent climate change, defining cause-and-effect relationships between temperature trends and biological behaviors (Gordo and Sanz 2005; Bonofiglio et al. 2009; Orlandi et al. 2012, 2016). In a study by Cerlini et al. (2021), temperature time series and phenological data were analyzed in two different willow species over the course of 14 years, and correlations between phenological trends and local temperature trends were confirmed. In particular, it was seen that the temperature increase observed in central Italy played a key role in controlling the phenological phases of the two willow species, and that the orographic characteristics of the phenological areas considered led to the shift in phenophase duration (from 5 to 20 days) (Cerlini et al. 2021).

Therefore, the purpose of our study was to determine and analyze the average development trends of some tree species over a 14-year period (2008–2021) by dividing the collected data of each species into cold and warm years groups to analyze their differences in behavior in relation to environmental conditions. The phenological study was conducted according to the “International Phenological Garden (IPG)” method and used as a tool to investigate climate and plants.

## Materials and methods

### Phenological gardens of Rieti and Pian di Rosce

Plants within the two gardens come from the same clonal selections to eliminate the genetic element as the causes of differences in developmental rate; thus, variations in phenological responses will be determined by environmental factors such as difference in latitude, soil, weather patterns (Orlandi et al. 2020), and altitude (Vitasse et al. 2010). In the present work, the chosen tree species were placed in two gardens, in the Rieti City Phenological Garden (named Rieti Base, at 380 m asl and with an area of about 2000 m^2^) and the Rieti Mountain Garden named Rieti “Pian di Rosce” (from now on “Rosce”), at 1050 m asl with an area of about 3000 m^2^. Plant species are derived from mother plants received from the German Meteorological Service, the European coordinator of phenological garden activities. Through the observations, made in the gardens, each biological phase of the plants was interpreted using the international conventional key “BBCH” to obtain values comparable with those in the literature (Chmielewski and Roetzer 2001; Meier 2001; Saska and Kuzovkina 2010; Orlandi et al. 2020). The BBCH scale, whose abbreviation comes from Biologische Bundesanstalt, Bundessortenamt and Chemical industry, is a decimal system designed to uniformly code similar phenological stages for both monocotyledonous and dicotyledonous species. Its structure allows it to enclose all existing scales, and it can also be used for all those species for which special scales are not currently available. The entire plant development cycle is divided into ten long-lived developmental stages, described by numbers 0 to 9 in ascending order. The scale is divided into primary and secondary stages of development. Each stage is indicated by a code consisting of two numbers. Primary growth stages are not sufficient to accurately define application or evaluation dates; therefore, secondary stages are used if precise time points or stages of plant development need to be indicated. Unlike the main growth stages, secondary states are defined as short developmental stages of the plant species that are passed in succession during the respective main growth stage. These are also coded using the digits 0 to 9. Combining the numbers for the main and secondary stages gives rise to the two-digit code (Meier 2001). The BBCH scale does not consider all phenophases but only those that are most agronomically important and correlated with data collected by Phenological Gardens throughout Europe. Specifically, in this work, the following phenological phases were considered: for the vegetative cycle, the stages BBCH_11 = first leave unfolded; BBCH_19 = 9 leaves unfolded; BBCH_91 = beginning of senescence stage, shoot development completed, but foliage still green; for the reproductive cycle, the stages BBCH_65 = full flowering: 50% of flowers open, first petals may have fallen off. To limit randomness, in each garden, observations were conducted on three individuals of each tree species by calculating the date of the appearance of each phenophase as the average of the three plants (Orlandi et al. 2020). To limit randomness, in each garden, observations were conducted on three individuals of each tree species by calculating the date of the appearance of each phenophase as the average of the three plants (Orlandi et al. 2020). Phenological observations for both vegetative and reproductive stages were realized weekly (almost always mid-week) recording any peculiarities of the phenological stages that might affect normal plant development.

In both phenological gardens, there are species common to both such as *Cornus mas.*, *Salix smithiana*, and *Salix viminalis*, and species not common to both; in the Rieti Garden, there are, in addition to those already mentioned, *Corylus avellana* and *Crataegus monogyna*, in Rosce; on the other hand, there are the species *Cornus sanguinea* and *Salix acutifolia*.

### Meteorological data methodology

Meteorological data for the two Phenological Gardens of Rieti and Rosce were provided by the relevant weather stations located in the vicinity of the Gardens and operated by the “Centro Appenninico del Terminillo” of the University of Rieti. Temperature records were considered, covering a 14-year period (2008–2021).

With the use of temperatures, GDH (growing degree hours) and GDD (growing degree days) values were calculated. With GDDs, “degree days” or “temperature sums” are calculated, which are based on the average daily temperature value, from which the temperature below which the species under consideration does not grow, was subtracted. The later in specific was calculated with temperature threshold 7 °C. GDD7 and GDH were related to the weeks, taking Wednesday as the reference day of the week, in which the phenological stages of interest occur for each species considered, to highlight the climatic variables influences on the result of physiological processes, e.g., leaf unfolding. Measuring the amount of heat accumulated by plants over time thus gives important insights into the rate of phenological development. Daily data for the 14-year period (2008–2021) were averaged to obtain weekly values.

### Trend analysis

Phenological trends were analyzed using nonparametric Mann–Kendall tests to identify positive or negative monotonic trends. Positive *Z* values reveal a tendency for phenological data to be delayed, while negative *Z* values indicate a tendency for phenological data to be advanced. Sen’s nonparametric method was used to estimate the slope of the regression line. The most significant values are highlighted by the following symbols:

 + , for a slope at 0.1; *, for a slope < 0.05; **, for a slope < 0.01; and ***, for a slope < 0.001.

Mann–Kendall trend analysis was performed with the Excel model application MAKESENS version 1.0 (Salmi et al. 2002; Orlandi et al. 2020).

### Correlation analysis

For each tree species, Pearson correlation analysis was conducted between the dates of occurrence of the phenological phases BBCH_11 and BBCH_65 and the monthly averages of maximum and minimum temperatures in January, February, and March to show possible relationships.

### Cold and warm groups

Through the definition of groups of “warm” (WG) and “cold” years (CG), we want to investigate the effect of climatic warming on the phenological behaviors of the studied species. The group of warm years (WG) can be useful to understand what the responses of the vegetative and reproductive development stages of plants subjected to temperature increases of a few degrees centigrade might be.

During 2008–2021, quarterly averages of daily maximum and minimum temperatures were calculated in relation to the period of occurrence of different phenological stages in the two study areas. For example, for the earliest stage (BBCH_11), the first quarter of the year (January, February, and March) was considered. A rather long period of 3 months was chosen to be considered to understand weather trends that may result in physiological developments in the observed plants from a phenological point of view as also highlighted in the literature (Montgomery et al. 2020). Then considering both maximum and minimum temperatures, medians of the quarterly averages were calculated, and years with values above and below the medians, respectively, were grouped together, thus identifying WG and CG. This division into groups is useful to highlight any differences in the behavior of the same species placed in relation between years with quarterly maximum temperatures higher or lower than the median. Following the division of the “cold” and “warm” groups, the GDD values with threshold value 7 °C (GDD7), and the number of the week (starting from Jan. 1) in which the analyzed phenological phase appears (BBCH_n) for each species, phenological phase and phenological garden were considered. Finally, the main parameters of descriptive statistics were derived from the described parameters: the mean, standard deviation, and coefficient of variation.

### Interpretation BBCH-GDD7 behaviors between warm and cold years

To interpret the different possible behaviors in response to the presence of warm and cold years, an interpretive matrix was constructed (Table 1) where one axis predicts the advance, delay, or constancy of the date of realization of the phenological phase while the other axis predicts the reduction, increase, or constancy of the warm requirement for the realization of the same phase expressed as heat storage.

**Table 1:**
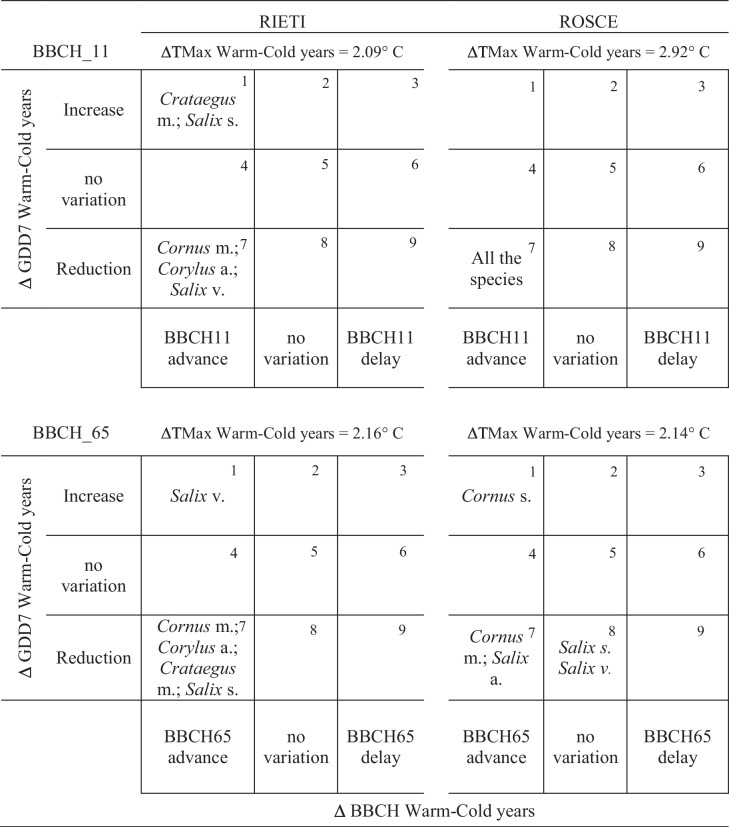
A plant species interpretive matrix in the “warm-cold” groups showing nine behavioral cases of phenological phases and temperature accumulations.

In this way, considering the differences in average temperatures between warm and cold groups (Δ WG-CG), the matrix predicts 9 hypothetical cases that can be summarized as follows:

- Case 1: In warm years (WG), anticipation of the phenological phase occurs due to higher heat demand, as if the plant species probably does not anticipate the realization of the phase so much as to maintain the same GDD7 accumulation.
- Case 2: In WG, there is a constancy in phenological phase realization but an increase in GDD7 accumulation, as if the plant species realizes the phase independently of accumulation.
- Cases 3–6-9 represent the hypotheses for which phenological stage delay would occur simultaneously with increases, not changes or reductions in GDD7 accumulations.
- Case 4: In WG, there is a constancy in heat accumulations and an advance in the phenological phase, as if the plant species realized the phase earlier but still with the same heat accumulations.
- Case 5: No change in behavior is manifested either as heat accumulation or phenological phase considering warm or cold years.
- Case 7: In WG, the advancement of the phenological phase occurs despite reduced heat demand, as if the plant species anticipates the realization of the phase more than proportionally to the accumulation of GDD7.
- Case 8: In WG against a constancy in phenological phase realization, there is a reduction in GDD7 accumulation, as if the plant species realizes the phase regardless of accumulation.

## Results

### Meteorological variable trend

**Fig. 1:**
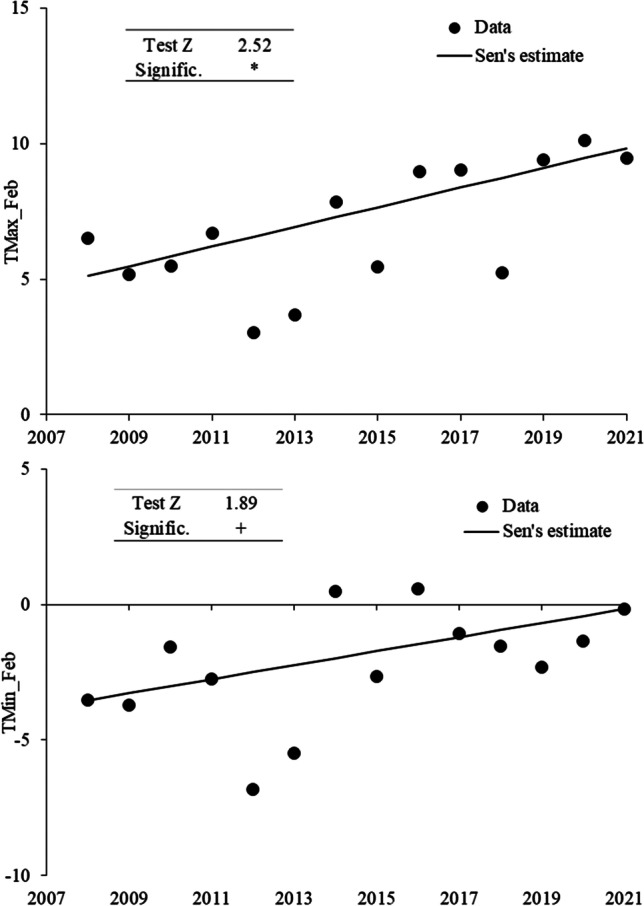
Meteorological variables trend of Rosce garden, TMax_Feb with Z-test. + *a* = 0.1; * *a* < 0.05; ** *a* < 0.01; *** *a* < 0.001

Considering daily meteorological values, the most significant trends over the study period were elaborated. Both maximum and minimum temperatures show significant trends in February at both Rosce and Rieti; in particular, in Fig. 1, the trends of TMax and TMin at the Rosce station are presented because of their greater significance (TMax test Z 2.52; TMin test Z 1.89) although similar to those found at Rieti.

### Variability of phenological dates and heat accumulations in the two gardens

After calculating in both gardens, the average dates of phenological phases and the corresponding heat accumulations for each species from 2008 to 2021, low CV values (always below 0.5) referring to the occurrence of all phenological phases, and their respective GDD7s are observable in the Rieti Garden (Table 2). Only the BBCH_65 phase shows highly variable values of the coefficients of variation with respect to the other phases. In particular, the CV of BBCH_65 dates shows a value greater than 1 in three out of five cases and in *Cornus m*., in *Corylus a.*, and in *Salix s*., respectively, while in the other species, it is between 0.88 and 0.99. This means that the date of occurrence of the BBCH_65 phase of the species in the Rieti garden has been highly variable during the time period in which they have been monitored. In Table 1, referring to the Rosce garden, the CV value of dates and GDD7 heat accumulation is always less than 0.5 in all species. In the case of GDH accumulations in BBCH_11, the CV is greater than 0.5 in all 5 species except for *Salix s*., and for BBCH_65, it is greater than 0.5 for all species except for *Cornus s*. in the Rosce garden. Comparing the two gardens, it appears that in Rieti, the species in common between the two gardens (*Cornus m.*, *Salix s.*, and *Salix v.*) anticipate the date of appearance of all phenological stages by an average of two to three weeks than in Rosce.

**Table 2:** Mean appearance week, from 2008 to 2021, of the BBCH phase and relative heat accumulations (GDD7 and GDH) of each species in Rieti and Rosce phenological gardens (respectively at 380 m and 1050 m asl)

RIETI		*Cornus m*		*Corylus a*		*Crataegus m*		*Salix s*		*Salix v*	
		Mean	CV	Mean	CV	Mean	CV	Mean	CV	Mean	CV
BBCH_11	Week	16	0.08	14	0.08	13	0.09	14	0.09	13	0.09
	GDD7	7864	0.20	8061	0.20	7318	0.21	7435	0.21	6950	0.20
	GDH	123.2	0.30	128.3	0.31	108.1	0.31	108.5	0.26	96.62	0.35
BBCH_19	Week	19	0.06	18	0.04	17	0.09	17	0.07	18	0.05
	GDD7	13,253	0.17	13,552	0.15	12,377	0.22	12,260	0.16	13,314	0.18
	GDH	267.8	0.26	288.6	0.20	246.1	0.32	242.8	0.19	278.5	0.25
BBCH_91	Week	22	0.07	22	0.11	21	0.08	20	0.10	22	0.07
	GDD7	18,921	0.20	20,863	0.27	20,506	0.21	17,969	0.22	22,090	0.18
	GDH	434.5	0.30	497.6	0.36	491.7	0.26	404.4	0.30	533.9	0.23
BBCH_65	Week	13	1.23	7	1.40	17	0.88	10	1.19	11	0.99
	GDD7	4403	0.22	3115	0.38	12,525	0.14	4618	0.20	5053	0.21
	GDH	41.88	0.46	23.48	0.74	256.3	0.22	45.45	0.40	51.86	0.45
ROSCE		*Cornus m*		*Salix a*		*Salix s*		*Salix v*		*Cornus s*	
		Mean	CV	Mean	CV	Mean	CV	Mean	CV	Mean	CV
BBCH_11	Week	17	0.07	16	0.09	15	0.05	15	0.11	16	0.09
	GDD7	6889	0.17	6715	0.23	5910	0.19	6122	0.22	6171	0.20
	GDH	55.41	0.59	52.87	0.57	32.3	0.42	39.7	0.71	38.52	0.56
BBCH_19	Week	22	0.05	20	0.05	19	0.07	20	0.05	21	0.07
	GDD7	11,733	0.20	10,249	0.12	8899	0.17	9953	0.16	10,822	0.21
	GDH	179.5	0.27	142.5	0.28	109.7	0.32	134.7	0.32	157.3	0.34
BBCH_91	Week	25	0.06	23	0.06	22	0.08	23	0.06	25	0.11
	GDD7	18,188	0.17	14,635	0.20	12,981	0.24	14,090	0.19	17,779	0.38
	GDH	379	0.29	267.3	0.36	227.8	0.41	250.2	0.35	399.5	0.58
BBCH_65	Week	13	0.12	13	0.13	11	0.15	13	0.12	25	0.03
	GDD7	4553	0.26	4709	0.27	3997	0.30	4644	0.26	16,667	0.12
	GDH	12.14	0.60	15.65	0.82	6.682	1.01	13.84	0.89	341.6	0.17

### Correlation analysis results

Phenological phases BBCH_11 and BBCH_65 show a strong correlation especially with temperatures in February (Table 3), the only month in which a significant trend emerged with rising maximum and minimum temperatures over the study period. The correlations, considering the TMax of February, are higher mainly for the species placed in the Rieti garden (*r* values between 0.7–0.8).

**Table 3:** Correlation coefficients between TMax and TMin from January to March and phenophases (BBCH_11 and BBCH_65) in Rieti and Rosce

	TMax			TMin		
Rieti	January	February	March	January	February	March
BBCH_11_*Cornus m*	0.36	− 0.70	− 0.31	0.39	− 0.27	0.22
BBCH_11_*Corylus a*	0.60	− 0.71	− 0.30	0.29	− 0.43	0.21
BBCH_11_*Crataegus m*	0.36	− 0.60	− 0.19	0.27	− 0.57	0.24
BBCH_11_*Salix s*	0.34	− 0,47	− 0.08	− 0.11	− 0.57	− 0.10
BBCH_11_*Salix v*	0.24	− 0.62	− 0.42	0.29	− 0.35	0.47
BBCH_65_C*ornus m*	− 0.06	− 0.80	− 0.30	0.10	− 0.63	0.07
BBCH_65_*Corylus a*	0.30	− 0.68	0.28	− 0.13	− 0.55	− 0.13
BBCH_65_*Crataegus m*	0.19	− 0.72	− 0.20	0.31	− 0.53	0.10
BBCH_65_*Salix s*	− 0.01	− 0.78	− 0.15	0.11	− 0.56	− 0.04
BBCH_65_*Salix v*	− 0.05	− 0.59	− 0.12	0.14	− 0.35	0.33
Rosce	January	February	March	January	February	March
BBCH_11_*Cornus m*	0.14	− 0.34	− 0.45	0.35	− 0.25	− 0.02
BBCH_11_*Cornus s*	− 0.15	− 0.19	− 0.38	0.21	− 0.10	− 0.12
BBCH_11_*Salix a*	− 0.17	− 0.55	− 0.21	− 0.08	− 0.43	0.42
BBCH_11_*Salix s*	− 0.42	− 0.57	− 0.07	− 0.04	− 0.53	0.19
BBCH_11_*Salix v*	− 0.38	− 0.32	− 0.23	− 0.06	0.01	0.27
BBCH_65_*Cornus m*	− 0.02	− 0.58	− 0.39	0.17	− 0.56	0.27
BBCH_65_*Cornus s*	− 0.27	− 0.37	− 0.03	0.10	− 0.38	0.21
BBCH_65_*Salix a*	− 0.10	− 0.51	− 0.26	− 0.03	− 0.27	0.58
BBCH_65_*Salix s*	− 0.32	− 0.21	0.07	− 0.33	− 0.03	0.53
BBCH_65_*Salix v*	− 0.15	− 0.44	− 0.19	0.01	− 0.13	0.51

### Phenological phase trends and heat accumulation

Regarding the trends of phenological phases and heat accumulations (GDD7 and GDH) related to the Rieti area (Table 4), we show, for phase BBCH_11, a negative trend in all species, except for *Salix s*. Regarding temperature accumulations, only those calculated by GDH show significant trends for the species *Crataegus m.*, *Salix s.*, and *Salix v*. For phenological phase BBCH_65, *Cornus m*. and *Salix v*. show significant advance in both gardens as also does *Crataegus m*., present only in Rieti.

**Table 4:** Phenological phase trends and relative heat accumulations for the Rieti and Rosce area

RIETI	*Corylus a*		*Cornus m*		*Crataegus m*		*Salix s*		*Salix v*	
	Test *Z*	Sig	Test *Z*	Sig	Test *Z*	Sig	Test *Z*	Sig	Test *Z*	Sig
BBCH_11	− 2.773	**	− 2.607	**	− 2.888	**	− 1.425		− 2.578	**
GDD7	− 0.985		− 1.423		− 1.423		− 0.985		− 1.752	+
GDH	− 0.603		− 0.932		− 2.248	*	− 2.029	*	− 2.358	*
BBCH_19	− 0.961		− 0.871		− 1.471		− 1.232		− 1.187	
GDD7	− 0.438		− 0.328		− 1.204		− 0.547		− 0.328	
GDH	0.164		− 0.493		− 1.59		− 1.151		− 0.274	
BBCH_91	− 0.057		− 0.566		0.506		1.01		− 0.056	
GDD7	0.109		− 0.109		0.657		1.095		− 0.219	
GDH	0		− 1.151		0.274		0.384		− 0.493	
BBCH_65	0		− 2.201	*	− 1.8	+	− 1.359		− 2.127	*
GDD7	1.204		− 0.328		− 1.423		0.109		− 0.219	
GDH	0.822		− 0.493		− 0.713		0		− 0.274	
ROSCE	*Cornus m*		*Cornus s*		*Salix a*		*Salix s*		*Salix v*	
	Test *Z*	Sig	Test *Z*	Sig	Test *Z*	Sig	Test *Z*	Sig	Test *Z*	Sig
BBCH_11	− 1.750	+	− 1.442		− 2.065	*	− 0.856		− 2.065	*
GDD7	− 0.876		− 1.533		− 0.985		− 0.985		− 0.985	
GDH	0.055		− 1.590		− 0.822		− 0.493		− 0.822	
BBCH_19	0.346		− 0.682		− 0.356		− 0.463		0.346	
GDD7	0.438		− 0.985		− 0.328		− 0.219		0.438	
GDH	0.384		− 0.932		0.055		0.274		0.384	
BBCH_91	0.000		0.168		− 1.126		0.553		0.397	
GDD7	0.438		0.219		− 0.219		0.219		0.657	
GDH	0.384		0.493		− 0.384		0.274		0.822	
BBCH_65	− 2.764	**	− 0.837		− 0.560		− 0.578		− 2.764	**
GDD7	− 1.642		0.547		− 0.328		− 0.657		− 1.642	
GDH	− 0.055		1.042		0.274		0.055		− 0.055	

+ *a* = 0.1; * *a* < 0.05; ** *a* < 0.01; *** *a* < 0.001

Also, for the garden of Pian di Rosce (Table 4), the most significant trends are shown for the phenological phase BBCH_11, more specifically concerning the species *Cornus m.*, *Salix a*., and *Salix v*., which also anticipate here the date of appearance of the phase (showing a negative value). The significance of the trend for phenological stage BBCH_65, negative in *Cornus m*. and *Salix v*., is evident (earlier phenophases dates).

### Differences between groups of cold and warm years for the different species

Table 5 shows the average values of the vegetative and reproductive stages, particularly BBCH_11 and BBCH_65, and heat accumulations (GDD7) that showed the most marked differences in behavior. The division of warm and cold year groups was selected by considering only the maximum temperatures, which allowed the same groups of years to be more clearly discriminated from each other. In details, not all the analyzed 14-years belong to the group of warm and cold years considering that some years were very close to the medians of the quarterly averages. In Rieti, both the BBCH_11 and BBCH_65 cold and warm groups were represented by 5 years each. In Rosce, the BBCH_11 cold group was represented by 5 years while the warm group by 4. The BBCH_65 cold group in Rosce included 5 years and the warm group had 6 years.

**Table 5:** Mean BBCH_11 and BBCH_65 with relative GDD7 calculated in Cold and Warm groups in Rieti and Rosce phenological gardens

		Rieti				Rosce			
		Cold group		Warm group		Cold group		Warm group	
		GDD7	BBCH (Week)	GDD7	BBCH (Week)	GDD7	BBCH (Week)	GDD7	BBCH (Week)
BBCH_11	*Cornus m*	7578	17.6	6242	15.8	7415	17.4	6685	15.8
	*Salix s*	6999	14	7479	13.0	6459	16.2	6154	15.0
	*Salix v*	6999	14	6737	12.0	6779	16.2	6087	14.8
BBCH_65	*Cornus m*	5517	13.8	3934	12.0	5517	13.8	3925	11.6
	*Salix s*	4870	11.2	4495	9.0	4362	11.0	3852	11.2
	*Salix v*	5191	11.6	5261	10.4	5091	12.8	4439	12.4

The species placed in the two gardens (*Cornus m.*, *Salix s.*, and *Salix v*.) were presented in Table 5 to highlight the differences in phase and GDD7 between the 2 groups considered.

In the BBCH_11 phase of the Rieti Garden, *Salix s.* accumulates more heat in warm years (WG) than in the cold years (CG). The BBCH_11 average date, considering all the species, calculated during warm years in the Rieti garden anticipates the corresponding average date during cold years of 1.6 weeks (13.60 vs. 15.20). In Rosce, considering the BBCH_11 phase, the WG for each species showed lower GDD7 values than the cold group. In addition, the date of phase appearance in the warm group is advanced by about 1.4 weeks (15.20 vs. 16.60) with the highest advance presented by *Cornus m*.

Regarding phase BBCH_65, in the Rieti Garden, *Cornus m*. has a difference in heat accumulation between WG and CG of about 1600 GDD7. The date of appearance of this phase is always anticipated by the warm group, in this case, by 1.8 weeks.

In the phenological garden of Rosce, the GDD7 values for *Cornus m.* remain equally high as in Rieti, a large difference results regarding the date of appearance of the BBCH_65 phase of that species, which is anticipated in WG by 2.2 weeks. The specie *Salix s.* instead delays from 11 weeks in the CG to 11.2 weeks in the WG. The average for the BBCH_65 phase appearance date of all species is 0.8 weeks between WG and CG.

All species in both gardens belonging to the warm groups always show an advance in the appearance of the phenological phase, excluding *Salix s.* of the warm group related to the BBCH_65 phase of Rosce.

### Interpretive matrix of cold-warm groups

To facilitate the interpretation of results for each plant species, an interpretive matrix of behavior in the “warm” and “cold” year groups was created, showing the results of the vegetative and reproductive phases and the heat accumulations that highlighted the most pronounced differences in behavior (Table 1).

In the Rieti garden, during the BBCH_11 phase, with a TMax difference between cold and warm years of 2.09 °C, there is an advance in the appearance of the phase for the tree species *Crataegus m.* and *Salix s*. simultaneously with an increase in GDD7 accumulation (case 1); while the species *Cornus m, Corylus a*., and *Salix v*., under these same conditions, despite the advance in the phase behave differently than the previous species, presenting a reduction in heat accumulation (case 7). At Rosce, where there is a ΔTMax between cold and warm years of 2.92 °C, all species in the phenological garden advance the phase and reduce accumulation (case 7).

Regarding the BBCH_65 phase, it is observed that in the Rieti garden only for the species *Salix v*., the advancement of the phenological phase occurs against an increase in heat requirement (case 1), while all other species advance the phase but reduce heat requirement (case 7). More significant differences are present in the Rosce garden, within which almost all species exhibit different behaviors, fitting in different cases. Among all tree species, the two willows (*Salix s.* and *Salix v*.) show consistency in phase realization, reducing GDD7 accumulation (case 8).

In general, the cases in which species from both gardens are mainly found are the first and seventh, and only in Rosce also occurs case 8, for phase BBCH_65. During warm years, most of these species appear to anticipate both phenological phases by reducing their heat requirements, except for a few species that instead increase them. In one specific case, on the other hand (case 8), the period in phase realization remains constant despite a reduction in GDD7 accumulation, as if the plant species realizes the phase regardless of its heat accumulation.

## Discussion

From the results obtained from the work, it could be suggested that the more variable heat accumulation values found in the Rosce garden, and the delay in the period of appearance of phenological stages, are due to the lower average temperatures than in the Rieti garden, Rosce being located at an altitude above 1000 m. In the Rieti garden, in fact, located at lower altitudes, less temperature variability was found despite more variability in the dates of occurrence of phases, probably due to a higher mean temperature. Within the phenological area of Rieti; moreover, a lengthening of the vegetative period is generally shown, namely, an anticipation of the BBCH_11 phase by about two weeks compared to Rosce, in contrast to what emerges in the literature (Vitasse et al. 2010), in which this type of behavior is shown for species always placed at higher altitudes.

In addition, not one of the species analyzed in our study shows the need to accumulate a greater amount of warmth during warm years to trigger the appearance of the first leaves (BBCH_11 phase), as shown in the 2020 Montgomery et al. study, and on the contrary, we find a greater amount of GDD7 during cold years, with the exception of *Salix s.* and *Salix v*. in the Rieti garden.

The effects of climate warming interpreted through the phenological behaviors of the studied species are represented by the interpretive matrix of cold-warm groups. Case 7, in which the plant species anticipates phase realization more than proportionally to GDD7 accumulation, appears to be the most represented. The behavior of the species belonging to this case could be explained by the increase of both maximum and minimum temperatures during some restricted periods (February), which results in a strong advance of the phenological phase and a consequent reduction of the total accumulation from the beginning of the year to the realization of the phases. This thesis can be supported by the fact that both Rieti and Rosce show high correlations between the vegetative and reproductive phases and February temperatures (better correlations were shown above all with TMax), although in Rosce, the number of species with *r* values > 0.5 was lower than in the Rieti garden. Plant species that fall into this case can be considered, according to these deductions, to be plastic because they are able to change the date of realization of the phenological phase based on the influence of a limited period of warmth induction and the total “temperature requirements” interpreted through the calculation of GDD7. The phenological phase, influenced by more concentrated periods (even of a few weeks) where we find the real phenomenon of temperature forcing, is so much earlier that it leads to a reduction in total heat accumulation and thus to its variation.

In case 1, in which *Crataegus m*. and *Salix s.* are included for phase BBCH_11, *Salix v*. for BBCH_65 in Rieti, and *Cornus s*. for BBCH_65 in Rosce, climatic warming (interpreted through the different behavior in the warm-cold year groups “Δ WG-CG”) always results in the advancement of the phenological phase but with an increase in warmth requirement as if the plant species anticipates the realization of the phase less than proportionally to the accumulation of GDD7. Warm years seem to have less influence on the phenological phase which although slightly earlier does not lead to a reduction in accumulations being in fact greater in warm years than in cold years.

Again, plant species could be considered plastic since they can change the date of realization of the phenological stage according to environmental changes and their “temperature requirements.”

Case 8 is presented by only 2 species (*Salix s*. and *Salix v*. for phase BBCH_65 in Rosce). In this case, climatic warming (“Δ WG-CG”) determines constancy in the realization of the phenological phase and a reduction in GDD7 accumulation, as if the plant species realize the flowering phase independently of accumulation. Therefore, it could be supposed to be “rigid” species regarding phenological manifestation. Probably the rising trend of temperatures recorded in February does not affect the calculation of heat accumulations, and the reproductive development of these species in the highest phenological garden could be influenced by other variables besides temperature, such as photoperiod, highlighted in previous studies (Jackson 2009; Ma et al. 2020). Moreover, another parameter to consider may be the beginning of the ontogenetic development of some weeks before even if the only visible signs are represented by the increase of the water content in the flower buds.

All the tree species in the two phenological gardens, with the exception of Salix s. and Salix v., could be evaluated as plants with plasticity, as they are able to adapt to climatic warming by resisting and changing the dates of the phenological phases BBCH_11 and BBCH_65. The purpose of the work was to study these phenological responses in order to understand which types of trees in the face of temperature increases might be able to survive and which might suffer so much that they fail to adapt.

## Conclusion

Studying the behaviors of tree species to climatic warming is useful to distinguish which plants could be more resilient to temperature increases expected in future years and thus adapt to their environment, and which ones fail to do so and thus could succumb to unexpected extreme events. Plants, being bioindicators, are able to respond to environmental changes through various manifestations and can indicate to us that something in the environment around us is changing. Analyzing the results obtained from the various studies, it might be interesting to create predictive models so as to understand in advance how plants will react in relation to climate change. Finally, it might also be useful to analyze these issues by focusing on restricted periods in which temperature increases occur (a few weeks) that may be crucial to the manifestation of phenological phases, rather than focusing only on long periods.

## Data Availability

The datasets generated and/or analyzed during the current study may be available from the corresponding author upon reasonable request.
